# Specific Marker Expression and Cell State of Schwann Cells during Culture *In Vitro*

**DOI:** 10.1371/journal.pone.0123278

**Published:** 2015-04-10

**Authors:** Zhangyin Liu, Yu-Qing Jin, Lulu Chen, Yang Wang, Xiaonan Yang, Jia Cheng, Wei Wu, Zuoliang Qi, Zunli Shen

**Affiliations:** 1 Department of Plastic and Reconstructive Surgery, Shanghai 1^st^ People’s Hospital, Shanghai Jiao Tong University School of Medicine, Shanghai, People’s Republic of China; 2 Trauma Center, Shanghai 1^st^ People’s Hospital, Shanghai Jiao Tong University School of Medicine, Shanghai, People’s Republic of China; 3 Plastic Surgery Hospital, Chinese Academy of Medical Sciences & Peking Union Medical College, Beijing, People’s Republic of China; 4 Department of Plastic and Reconstructive Surgery, Shanghai 9^th^ People's Hospital, Shanghai Jiao Tong University School of Medicine, Shanghai, People’s Republic of China; Centro Cardiologico Monzino, ITALY

## Abstract

Schwann cells (SCs) in animals exist in different developmental stages or wound repair phases, distinguished mainly by the expression of SC-specific markers. No study has yet determined SC state under *in vitro* culture conditions, and the specific markers expressed in SC are obscure as well. In this study, we harvested sciatic nerves from newborn mice and isolated SCs by an enzyme-digestion method, then we examined the expression profiles of ten markers (S100, p75^NTR^, Sox10, Sox2, GAP43, NCAM, Krox20, Oct6, MBP, and MPZ) at both the RNA and protein levels in *in vitro* mouse SCs and speculated their relation with *in vivo* SC stages. We assayed RNA and protein levels of SC specific markers by immunofluorescence, Western Blot, and real-time quantitative RT-PCR. The results show that the expression of most markers (S100, p75^NTR^, GAP43, NCAM, Krox20, Oct6, MBP and MPZ) was not detectable in all of early stage cultured SCs. The expression of transcription factors Sox10 and Sox2 was, however, detectable in all SCs. After 8 days, the positive expression rate of all markers except GAP43 and Oct6 was almost 100%.These results indicates Sox10 is a necessary marker for SC identification, while S100 is not reliable. SCs cultured *in vitro* express Sox2, P75^NTR^, NCAM, GAP43, Oct6, and MPZ, suggesting that they are similar to *in vivo* undifferentiated iSCs or dedifferentiated iSCs after nerve injury.

## Introduction

Schwann cells (SCs), which surround nerve fibers in the normal peripheral nerve tissue, provide structure support, conduct nervous impulses along axons, clear debris after peripheral nerve axon damage, and guide axonal regeneration [1]. Currently, three methods, including cell morphology, specific markers, and co-culture with dorsal root ganglion neurons, are usually applied to identify SCs. Among them, specific marker is the most important indicator for identifying SCs or stem cell derived Schwann cell—like cells.

Previous studies have reported several SC-specific markers, and the most commonly used are S100 [2], MBP [1,3], MPZ [4,5], P75^NTR^[6,7], GFAP [6,8], NCAM [5,9], GAP43 [6,10], PMP22 [11], Sox10 [12], Oct6 [13], O4 [14], Krox20 [15] and Sox2 [5]. Among them, S100 and P75^NTR^ are the most frequently used markers for the identification of stem cell—derived Schwann cell—like cells [16–18]. However, because SCs are not a homogenous population, identifying *in vitro* cultured SC or stem cell derived Schwann cell—like cells based on a single or a few specific markers may not be a reliable method.

In animals, SC has various developmental stages or wound repair phases, and each of these has distinct specific markers [19]. SCs are originated from neural crest cells, which are further differentiated into Schwann cell precursors (SCPs) and immature Schwann cells (iSCs) during embryonic development. After the pro-myelin Schwann cell (pro-mSC) stage, two matured SCs are finally formed, which are the myelinating Schwann cell (mSC) and the non-myelinating Schwann cell (nmSC) [19]. After peripheral nerves are injured, mature SCs in the distal stump will dedifferentiate into iSCs, which will differentiate into mSCs and nmSCs after nerve regeneration [20–22].

The SC specific markers in various SC stages are different but partially overlapping. A previous study suggested that Sox10 may be the only known marker constitutively expressed in the whole SC development process [19,23], while S100, the widely used SC marker, is not expressed in SCPs [24]. In addition, when iSCs differentiate into mSCs, transcription factor Oct6 is first expressed in pro-mSCs, inducing the expression of downstream transcription factor Krox20, which in turn regulates the expression of myelin-associated genes MBP and MPZ [3,10,15,25–27]. Another study suggested that Sox2, the marker for undifferentiated stem cells, is only expressed in SCPs and iSCs [5,28,29]. In addition, P75^NTR^ and NCAM are both expressed in iSCs and nmSCs [5,6,8], while GAP43 is not only expressed in iSCs and nmSCs, but also in SCPs [1,6]. The expression pattern of common *in vivo* SC markers is summarized in Table 1.

**Table 1:** Special markers of *in vivo* Schwann cells.

Schwann cell stage	Markers
Schwann cell precursor (SCP)	Sox10, GAP43, Oct6, Sox2, MPZ
immature Schwann cell (iSC)	Sox10, S100, GAP43, P75^NTR^, NCAM, Sox2, Oct6, MPZ
pro-myelin Schwann cell (pro-mSC)	Sox10, S100, Krox20, Oct6
myelinating Schwann cell (mSC)	Sox10, S100, Krox20, Oct6 (early mSCs), MBP, MPZ
non-myelinating Schwann cell (nmSC)	Sox10, S100, GAP43, P75^NTR^, NCAM, Oct6 (low level)

Given that the expression of SC markers is dynamic *in vivo*, we wanted to know the expression profile of *in vitro* cultured SCs and to understand its relation with *in vivo* SC stages. However, in our literature review we did not find any relevant study addressing this question.

The present study covers 10 common SC markers (S100, P75^NTR^, Sox10, Sox2, GAP43, NCAM, Oct6, Krox20, MBP, and MPZ) and attempts to draw a rough marker map of SCs cultured *in vitro*. We observed cell morphology; generated the expression profiles of SC markers of *in vitro* and *in vivo* conditions by immunofluorescence, Western Blot and real-time quantitative RT-PCR methods; and attempted to determine the state of *in vitro* SC. Our data indicated that cultured SCs gradually shift from a heterogeneous state into one that is similar to *in vivo* iSC. In addition, we found that the commonly used markers S100 and P75^NTR^ were not expressed in the early stage of cultured SCs, while transcription factor Sox10 is expressed in all stages of cultured SCs, suggesting that S100 and P75^NTR^ may not be suitable for the identification of early SCs.

## Materials and Methods

### Animals

Newborn (5–7 days old) and adult (8–12 weeks old) C57BL/6 mice were purchased from Shanghai SLAC Laboratory Animal Co. Ltd. All animal protocols were approved by the Animal Experiment and Care Committee of Shanghai Jiao Tong University School of Medicine.

### SC isolation purification and enrichment

Newborn mice were sacrificed by decapitation, and their sciatic nerve (SN) segments were harvested aseptically under a dissecting microscope. Nerve segments were digested by an enzymatic solution containing Collagenase NB 4 (0.26 U/ml, Serva, Germany) and Dispase II(neutral protease, grade II, 0.94 U/ml, Roche, USA), which were both dissolved into DMEM (Thermo, USA) at a concentration of 0.2%.

To digest tissues, the enzymatic solution (50 μl per segment) was added and incubated at 37°C for 80 min within a cell incubator (Thermo, USA). The mixture was then centrifuged at 600×g for 10 min. After removal of the supernatant, the cell pellet was resuspended in Schwann cell culture medium (SCCM) consisting of DMEM medium supplemented with b-FGF 0.1 mg/ml (Peprotech, USA), heregulin-β-1 0.1 mg/ml (Peprotech, USA), 2 μM forskolin (Cayman,USA), 10% fetal bovine serum (FBS) (Hyclone, Australia), Penicillin-Streptomycin (Gibco, USA) [30,31]. The cells were cultured in a cell incubator with a humidified atmosphere of 5% carbon dioxide at 37°C.

After 48 hours, SCs were purified and enriched as described [31]. In short, after cultured 48 hours, the culture medium was replaced with 0.2% Dispase II diluted in DMEM with a volume of 0.1 ml/m^2^. After incubation at 37°C for 20 mins, the flasks were kept shaking horizontally for 1–3 min to release detaching cells. The suspended cells were then collected into a 15-ml centrifuge tube(BD Falcon, USA) and centrifuged at 600×g for 5min. After removal of supernatant, the pellet was resuspended in SCCM and plated onto flasks at a density of 2–2.5 × 10^4^/cm^2^. This purification and enrichment was repeated every 48 hours until the Schwann cells were cultured for a total of 8 days *in vitro*.

### Tissue Preparation

Mice were anesthetized with pentobarbitone and perfused through the left ventricle with 4% (w/v) paraformaldehyde (PFA, Sigma, USA) in phosphate-buffered saline (PBS) (pH 7.4). Unfixed tissue was used for real-time quantitative RT-PCR. The SN tissue was dissected, postfixed in 4% PFA and 10% (w/v) sucrose at 4°C for 2 hours, cryo-preserved in 30% (w/v) sucrose at 4°C overnight, embedded in O.C.T. compound, sectioned in a cryotome (10 μm). Longitudinal cryo-sections were thaw-mounted onto slides and stored at -20°C.

### Immunohistochemistry

Frozen sections, after several rinses in PBS, were permeabilized and blocked with PBS containing 0.3% (v/v) Triton X-100 and 10% (v/v) goat (or rabbit) serum for 30 mins at 37°C, then incubated at 4°C overnight with primary antibodies(without primary antibodies as blank control) followed by incubation with secondary antibodies for 40 mins at 37°C. Primary antibodies were diluted in PBS containing 0.1% Triton X-100,and secondary antibodies were diluted in PBS. Cell nuclei were counterstained with 4’,6-diamidino-2-phenylindole, dihydrochloride (DAPI, Invitrogen, USA) diluted in PBS(1:500). Slides were observed under a confocal laser scanning microscope (TCS SP5, Leica, Germany).

### Immunocytochemistry

Cells cultured for 2 days (at the primary passage, P0) and 8 days (at the third passage, P3) were fixed with 4% PFA in PBS at 37°C for 15 mins. Unfixed cells were used for Western blotting and real-time quantitative RT-PCR. Fixed cells, after several rinses in PBS, were permeabilized and blocked with PBS containing 0.3% Triton X-100 and 10% goat (or rabbit) serum in PBS for 30 mins at 37°C, then incubated at 4°C overnight with primary antibodies(without primary antibodies as blank control) followed by incubation with fluorophore-conjugated secondary antibodies for 30 mins at 37°C. Primary and secondary antibodies were diluted in PBS. Cell nuclei were counterstained with DAPI diluted in PBS(1:500). Cells were observed under a fluorescence microscope (Olympus, Japan).

### Western blotting

For Western immunoblotting, cultured cells were homogenized in lysis buffer (0.25 M Tris-HCl, pH 6.8, 20% glycerol, 4% SDS, 10% mercaptoethanol). The protein lysates were fractionated on a SDS-PAGE gel and transferred to nitrocellulose membrane (Millipore, USA). The blotted membranes were blocked with 5% nonfat milk in Tris-buffered saline containing 0.1% Tween-20 (TBST) at room temperature for 1 hour, and the membranes were incubated overnight at 4°C with primary antibodies diluted in prepared TBST containing 3% nonfat milk. After 3 washes in TBST, the blots were reacted with horseradish peroxidase-conjugated secondary antibodies (Abmart, China) for 1 hour at room temperature and then washed again with TBST. For detection, an enhanced chemiluminescence-Western blot system (Amersham, Piscataway, USA) was used. For quantification, the X-ray films were put through a scanner (Samsung, Seoul, Korea) and analyzed with LAS image analysis system (Fujifilm, Tokyo, Japan). The picture were analysed with software ImageJ (National institutes of Health, USA) to obtain gray value of each band. Then relative gray value of each marker(gray value of special marker / gray value of β-actin)were calculated and analysed.

### Primary and secondary antibodies

The following primary antibodies were used: anti-MBP (rabbit 1:250) (Abcam, UK), anti-Sox10 (rabbit 1:200) (Abcam, UK), anti-MPZ (rabbit 1:250) (Abcam, UK), anti-GAP43 (rabbit 1:250) (Abcam, UK), anti-P75 NGF Receptor (rabbit 1:500, (Abcam, UK), anti-Neural Cell Adhesion Molecule (rabbit 1:500) (Millipore, USA),anti-S100 (rabbit 1:500) (DakoCytomation, USA), Krox20 (rabbit 1:100) (Covance, USA), anti-Sox2 (rabbit 1:200) (Epitomics,USA), anti-Oct6 (goat 1:100) (Santa Cruz, USA), anti-β-actin (Abmart, China). For immunohistochemistry and immunocytochemistry tests, the secondary antibodies were conjugated with Alexa 488 and Alexa 555 (goat and rabbit 1:500) (Invitrogen, USA).

### Positive rate of SC markers

After immunostaining with antibodies, SCs and fibroblasts were identified based on their morphologies. Cells with a bipolar or tripolar shape were recognized as SCs, whereas flat or polygonal cells were recognized as fibroblasts [32,33]. The positive rate of SCs markers was derived from the calculation of the percentage of marker-positive SCs with respect to the total number of counted SCs:

$$\text{SC marker positive rate=}\frac{\text{Marker-positive SC number}}{\text{Total SC number}}\times \text{100\%}$$

We counted at least 100 SCs from at least 3 random photo-areas at 100x magnification for each experiment. Three independent experiments were repeated and Schwann cells in each experiment were harvested from a single isolation respectively.

### Real-Time quantitavtive RT-PCR

Total mRNAs using Trizol (Invitrogen, USA) were extracted from cultured cells and sciatic nerve tissue. Real-time quantitative RT-PCR was performed on the StepOnePlus instrument (Applied Biosystems, USA), using Promega M-MLV kit (Promega, USA) and SYBR green method. We set 40 cycles as a cycle cut-off point for no detection.

The primer sequences (designed by Invitrogen, USA) are listed below:

β-actin F 5'-CCTCTATGCCAACACAGT-3'
β-actin R 5'-AGCCACCAATCCACACAG-3'
S100 F 5'-GCTGAGCAAGAAAGAACTGAA-3'
S100 R 5'-AGCCACCAGCACAACATAC-3'
P75^NTR^ F 5'-GGTGATGGCAACCTCTACAGT 3'
P75^NTR^ R 5'-CCTCGTGGGTAAAGGAGTCTA-3'
Sox10 F 5'-AGATCCAGTTCCGTGTCAATAA-3'
Sox10 R 5'-GCGAGAAGAAGGCTAGGTG-3'
GAP43 F 5'-AAGGCAGGGGAAGATACCAC-3'
GAP43 R 5'-TTGTTCAATCTTTTGGTCCTCAT-3'
Krox20 F 5'-GCCCCTTTGACCAGATGAAC-3'
Krox20 R 5'-GGAGAATTTGCCCATGTAAGTG-3'
MBP F 5'-AGAGTCCGACGAGCTTCAGA-3'
MBP R 5'-CAGGTACTTGGATCGCTGTG-3'
MPZ F 5'-TCTCAGGTCACGCTCTATGTC-3'
MPZ R 5'-GCCAGCAGTACCGAATCAG-3'
NCAM F 5'-GGGAGGATGCTGTGATTGTCT-3'
NCAM R 5'-GCAGGTAGTTGTTGGACAGGAC-3'
Oct6 F 5'-CTCCTGGGGTCCTTCTAACT-3'
Oct6 R 5'-TTATACACAGATGCGGCTCTC-3'
Sox2 F 5'-GCTGGACTGCGAACTGGA-3'
Sox2 R 5'-GCGTTAATTTGGATGGGATTG-3'

For quantification we used β-actin as a calibrator for samples (nerve tissue or cultured SCs) to which unknowns could be compared. Newborn sciatic nerve or P0 SCs were a control. We compared the unknown samples against the calibrator sample to give relative gene expression using the Comparative C_T_ method 2^-meCT^ [34].

### Statistics

We repeated three independent experiments and Schwann cells in each experiment were harvested from a single isolation respectively. The positive rate of SC markers, results of real-time quantitative RT-PCR and gray value of Western blotting were presented in texts as mean values ±SD. For quantitative comparison and analysis, the values were subjected to Student’s *t*-test, significant at *P* < 0.05.

## Results and Discussion

### The *in vivo* expression profile of SC-specific markers

**Fig 1:**
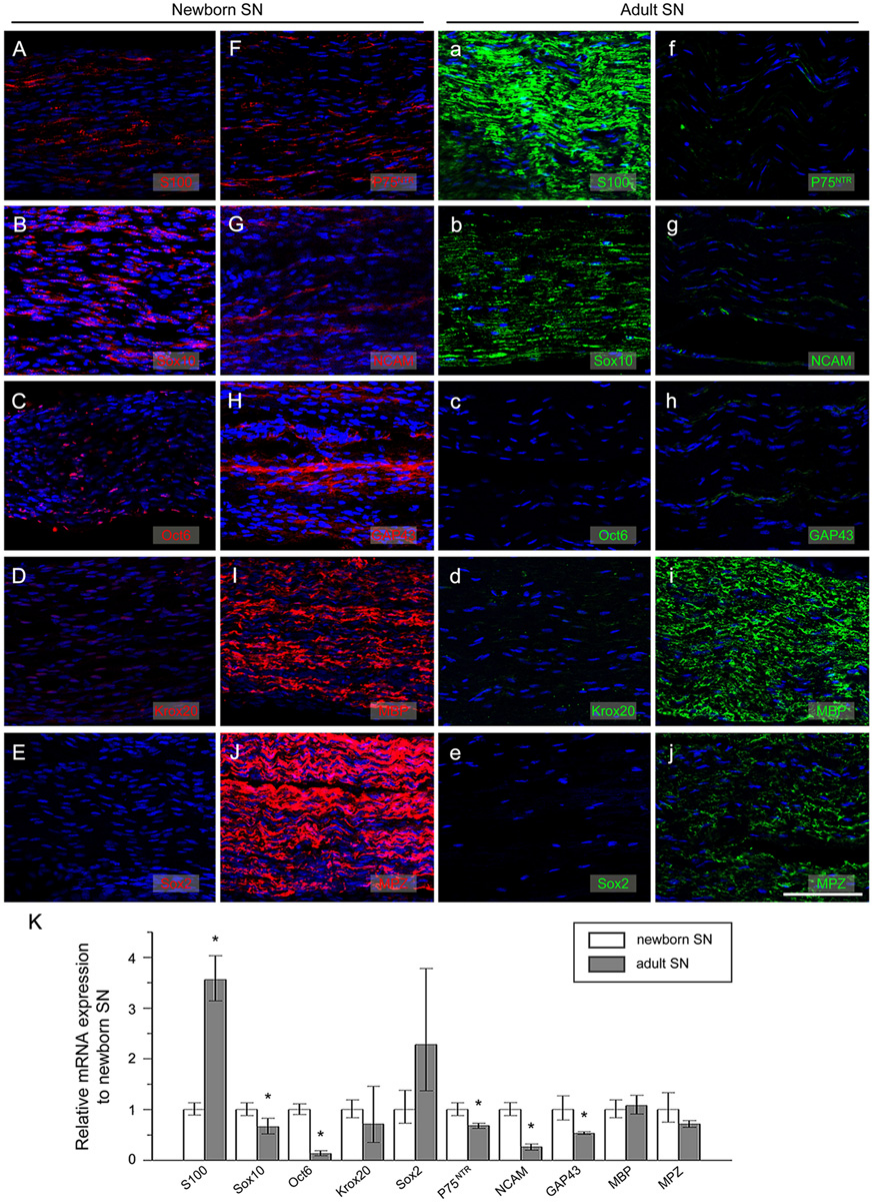
The expression of Schwann cell markers in murine sciatic nerve. Immunofluorescence staining of sciatic nerve vertical section from newborn (A-J) and adult (a-j) mice. (A, a) S100; (B, b) Sox10; (C, c)Oct6 failed to be detected in adult SN whereas newborn SN is Oct6 positive; (D, d) Krox20; (E, e)Sox2 was not expressed in both newborn and adult SN; (F, f) P75^NTR^; (G, g)NCAM; (H, h)GAP43; (I, i) MBP; (J, j) MPZ. (K) Real-time quantitative RT-PCR analysis of mRNA expression in newborn and adult mice SN. S100 mRNA level in adult SN increased while that of Sox10, Oct6, P75^NTR^, NCAM, GAP43 decreased during culture *in vitro*. *P<0.05. Scale bar = 100μm.

To determine the expression of SC-specific markers in SN derived from newborn and adult mice, we first performed immunofluorescence assays in SN from mice of different ages. We found a significant amount of nuclei in the SN from the newborn mice, but less in the SN from the adult mice. Although expression of Sox2 was undetectable in the SN from the newborn mice (Fig 1E), expression of the other 9 markers could be detected (Fig 1A–1D and 1F–1J). In the SN from the adult mice, we found no expression of Sox2 (Fig 1e) and only weak expression of Oct6 (Fig 1c), while the other 8 markers were detected with slight differences in their expression levels.

In addition, we compared the mRNA level of SC-specific markers in SN from the newborn and adult mice by real-time quantitative RT-PCR assay and statistical analysis (Fig 1K). We found that the mRNA level of S100 in the adult SN was higher than that in SN from the newborn mice, while the mRNA levels of Sox10, Oct6, P75^NTR^, NCAM and GAP43 in the adult SN were less than those in SN of newborn mice, suggesting a similar pattern between the mRNA level and the protein level.

### The *in vitro* expression profile of SC-specific markers

**Fig 2:**
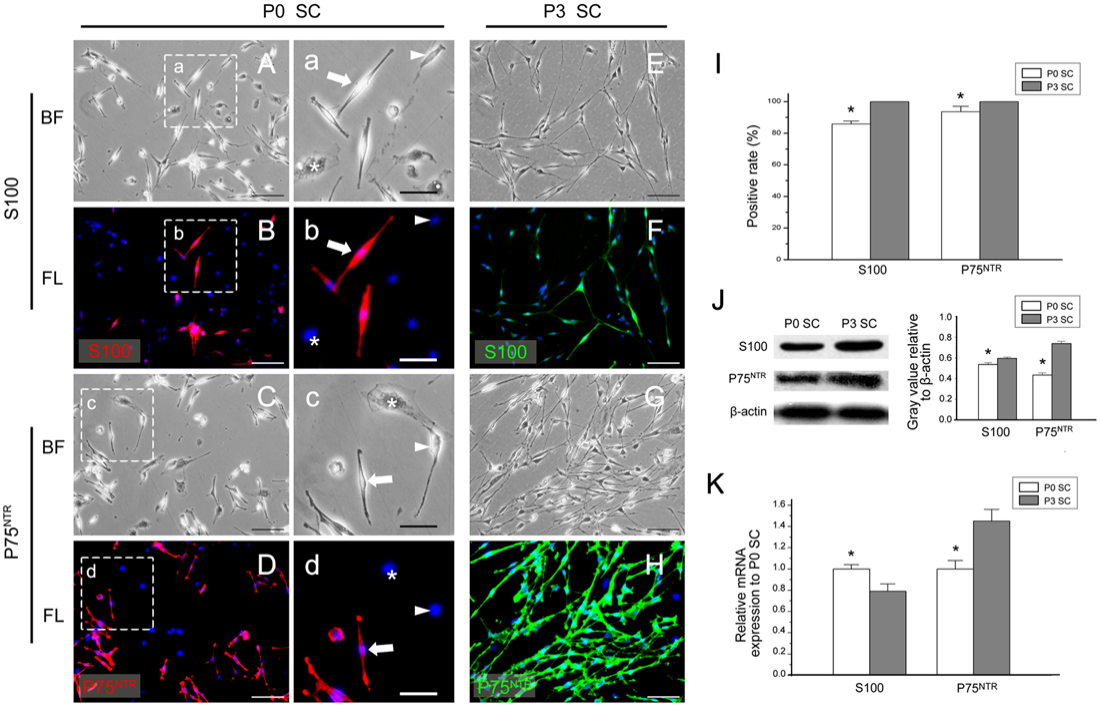
The expression of S100 and P75^NTR^ in *in vitro* cultured SCs. (A-H, a-d) The expression of S100 and P75^NTR^ in *in vitro* cultured SCs detected by immunofluorescence. SCs had a bipolar or tripolar spindle shape with a small cytoplasm / nuclear ratio and strong refraction rate (arrow, arrowhead), and fibroblast cells were flat and irregularly shaped with a large cytoplasm / nuclear ratio and poor refraction rate. In P0 SCs, a portion of SCs expressed S100 and P75^NTR^ (a-d arrow) while the rest of the SCs (a-d arrowhead) and all fibroblast cells (a-d asterisk) were negative for both markers. In P3 SCs, all SCs expressed S100 and P75^NTR^ (E-H). The bright-field (A, C, E, G, a, c) and immunofluorescence channel (B, D, F, H, b, d) are displayed. (I) The positive expression rate of S100 and P75^NTR^ in P0 SCs and P3 SCs. (J) The protein levels of S100 and P75^NTR^ in P0 SCs and P3 SCs were detected by Western blotting.(K) The mRNA levels of S100 and P75^NTR^ were determined by real-time quantitative RT-PCR analysis. The mRNA level of S100 was higher in P0 SCs, while the mRNA level of P75^NTR^ was higher in P3 SCs. (A-H) bar = 100μm, (a-d) bar = 50μm. * *P*<0.05.

After checking the *in vivo* expression of SC markers in SNs, we digested SNs harvested from the newborn mice by enzyme solution, and isolated cells for *in vitro* culture. We observed two kinds of distinct cell morphology under light microscopy: some cells had a bipolar or tripolar spindle shape with a small cytoplasm / nuclear ratio and a strong refraction rate (Fig 2a and 2c, arrow, arrowhead), which are consistent with the morphology of SCs [35]. In contrast, other cells were flat and irregularly shaped with a large cytoplasm / nuclear ratio and a poor refraction rate (Fig 2a and 2c asterisk), which were recognized as fibroblasts [35]. After three rounds of purification and culture, the obtained SCs had very little contamination from fibroblasts (Fig 2E).

### The *in vitro* expression of S100 and P75^NTR^, classic SC markers

We first checked the expression levels of S100 and P75^NTR^, the most widely used SC markers in the field of neuron development and regeneration, in *in vitro* cultured SCs. Immunofluorescence staining data showed that in the early stage of SC culture SCs didn’t totally express S100 and P75^NTR^ (Fig 2a–2d arrow), and some SCs could not express either marker (Fig 2a–2d, arrowhead), which show a staining pattern similar to fibroblasts (Fig 2a–2d, asterisks). After three passage culture, all SCs expressed S100 and P75^NTR^ (Fig 2E–2H). In P0 SCs, the early stage of cultured cells, the positive rates for S100 and P75^NTR^ were 85.84% ± 1.86% and 93.57% ± 3.40%, respectively. Interestingly, the positive rates for both markers reached 100% in P3 cultured SC (Fig 2I). After extracting total proteins from SCs at different time points and assaying by Western blot, we found that the protein levels of S100 in P3 SCs were higher than those in P0 SCs (Fig 2J). The grey value of S100 in P0 SCs and P3 SCs were 0.540 ± 0.014 and 0.596 ± 0.012. While the protein levels of P75^NTR^ in P3 SCs were significantly higher than those in P0 SCs, which grey value in P0 SCs and P3 SCs were 0.435 ± 0.020 and 0.738 ± 0.023, respectively. Real-time quantitative RT-PCR data showed that the mRNA level of S100 in P0 SCs was significantly higher than that in P3 SCs, while the opposite trend was detected in the mRNA level of P75^NTR^ (Fig 2K).

### The expression profile of transcription factors Sox10, Sox2, Oct6 and Krox20

**Fig 3:**
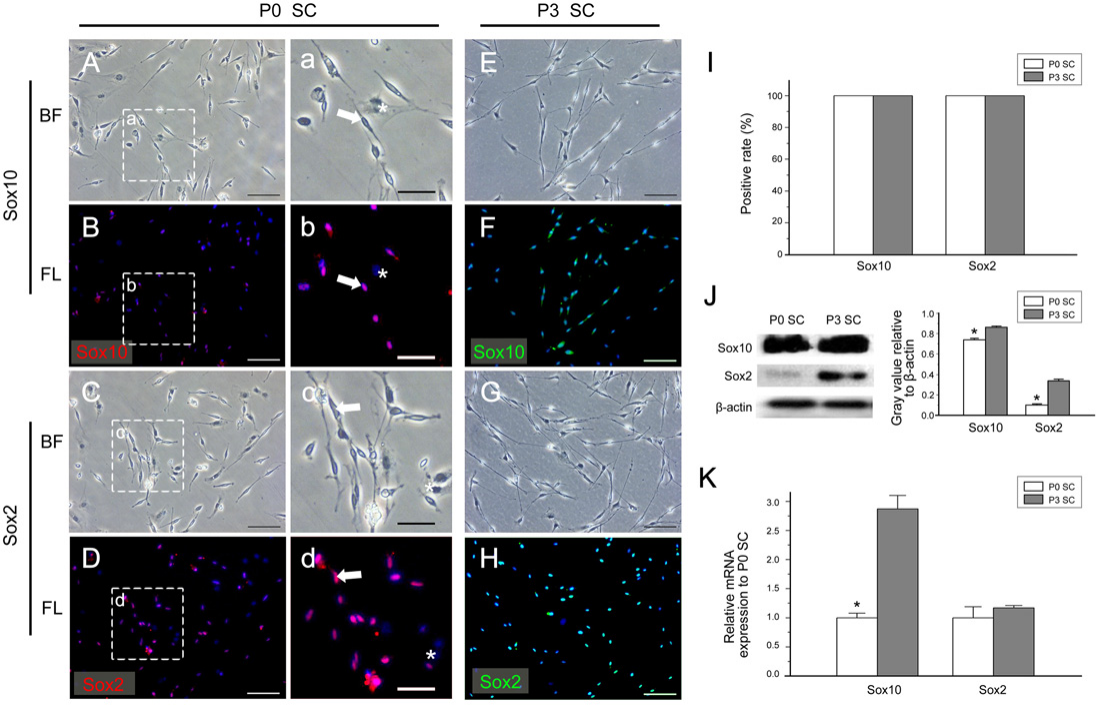
The expression of Sox10 and Sox2 in cultured SCs. (A-H, a-d) The expression of Sox10 and Sox2 in *in vitro* cultured SCs detected by immunofluorescence. In P0 SCs, all SCs are Sox10^+^/Sox2^+^ (a-d arrow), while all fibroblasts are negative (a-d asterisk). In P3 SCs, Sox10 and Sox2 were stably expressed in all SCs (E-H). The bright-field (A, C, E, G, a, c) and immunofluorescence channels (B, D, F, J, b, d) are displayed. (I) The positive expression rate of Sox10 and Sox2 in P0 SCs and P3 SCs. (J) The protein levels of Sox10 and Sox2 in P0 SCs and P3 SCs were detected by Western blotting. (K) The mRNA levels of Sox10 and Sox2 were determined by real-time quantitative RT-PCR analysis. The mRNA level of Sox10 was higher in P3 SCs than that in P0 SCs, while no significant difference in the mRNA level of Sox2 was detected. Bar = 100μm (A-H), bar = 50μm (a-d). * *P* <0.05.

We also detected the expression of Sox10, Sox2, Oct6 and Krox20, four transcription factors involved in regulating the development, differentiation, and myelination of SCs [1,19,36,37]. We found that Sox10 and Sox2 are constitutively expressed in all SCs with 100% expression rate (Fig 3a–3d arrow, Fig 3I), but not in fibroblasts (Fig 3a–3d asterisk). The protein levels of Sox10 and Sox2 were higher in P3 SCs than those in P0 SCs (Fig 3J). The grey value of Sox10 in P0 SCs and P3 SCs were 0.738 ± 0.013 and 0.863 ± 0.011, and the grey value of Sox2 were 0.098 ± 0.012 and 0.341 ± 0.013.

**Fig 4:**
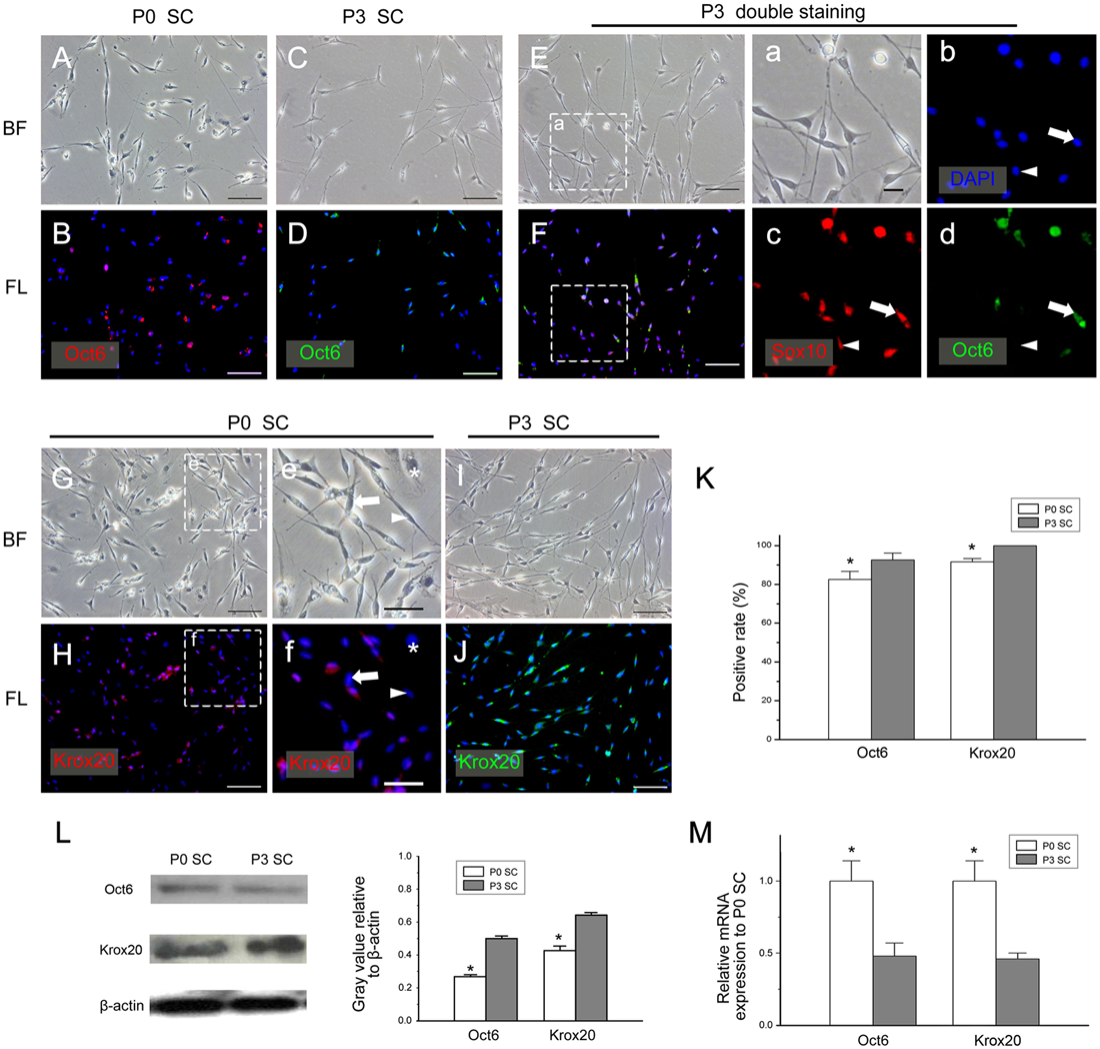
The expression of Oct6 and Krox20 in *in vitro* cultured SCs. (A-F, a-d) The expression of Oct6 was detected by immunofluorescence. In P0 SCs and P3 SCs, some SCs did not express Oct6 (A-D). The P3 SCs were double stained with Sox10 and Oct6 antibodies (E, F, a-d). Most SCs were Sox10^+^ / Oct6^+^ (a-d arrow), and a small part of the SCs were Sox10^+^ / Oct6^-^ (a-d arrowhead), while fibroblasts (E, F asterisk) were double negative. In P0 SCs, there were Krox20^+^SCs (e, f arrow) and Krox20^-^ SCs (e, f arrowhead). Fibroblasts were Krox20-negative (e, f asterisk). In P3 SCs, all SCs expressed Krox20 (I, J). The bright-field (A, C, E, G, I, a, c, e) and the immunofluorescence channels (B, D, F, H, J, b-d, f) are displayed. (K) The positive expression rate of Oct6 and Krox20 in P0 SCs and P3 SCs.(L) The protein levels of Oct6 and Krox20 in P0 SCs and P3 SCs were detected by Western blotting. The protein levels of both markers were higher in P3 SCs than those in P0 SCs. (M) The mRNA levels of Oct6 and Krox20 were determined by real-time quantitative RT-PCR analysis. (A-J) bar = 100μm, (a-f) bar = 50μm. *P <0.05.

The mRNA level of Sox10 in P3 SCs was higher than that in P0 SCs, while no significant difference in the mRNA level of Sox2 was detected at different culture times (Fig 3K). Surprisingly, the transcription factor Oct6 were not expressed in all P0 and P3 SCs (Fig 4), and the positive rate was 82.59% ± 4.13% and 92.64% ± 3.49%, respectively (Fig 4K). To further verify this result, we did Sox10 and Oct6 double staining in P3 SCs (Fig 4E, 4F, 4a–4d) and found that most SCs were Sox10^+^ / Oct6^+^, while the rest part of SCs were Sox10^+^ / Oct6^-^. In contrast, fibroblasts were all Sox10^-^ / Oct6^-^ (Fig 4E and 4F).

Furthermore, the expression of Krox20, the downstream transcription factor of Oct6, was similar to that of S100. It is not expressed in all in early *in vitro* cultured SCs (Fig 4e and 4f arrowhead), and the positive expression rate is approximately 91.67% ± 1.71% (Fig 4K). After cultured for 8 days, Krox20 is expressed in all SCs. The protein levels of Oct6 and Krox20 in P3 SCs was higher than those in P0 SCs (Fig 4L), and the grey value of Oct6 in P0 SCs and P3 SCs were 0.269 ± 0.011 and 0.501 ± 0.014, and the grey value of Krox20 were 0.428 ± 0.025and 0.644 ± 0.012. And the similar pattern was observed in the mRNA content of these two transcription factors (Fig 4M).

### The expression of myelin-associated protein MBP and MPZ

**Fig 5:**
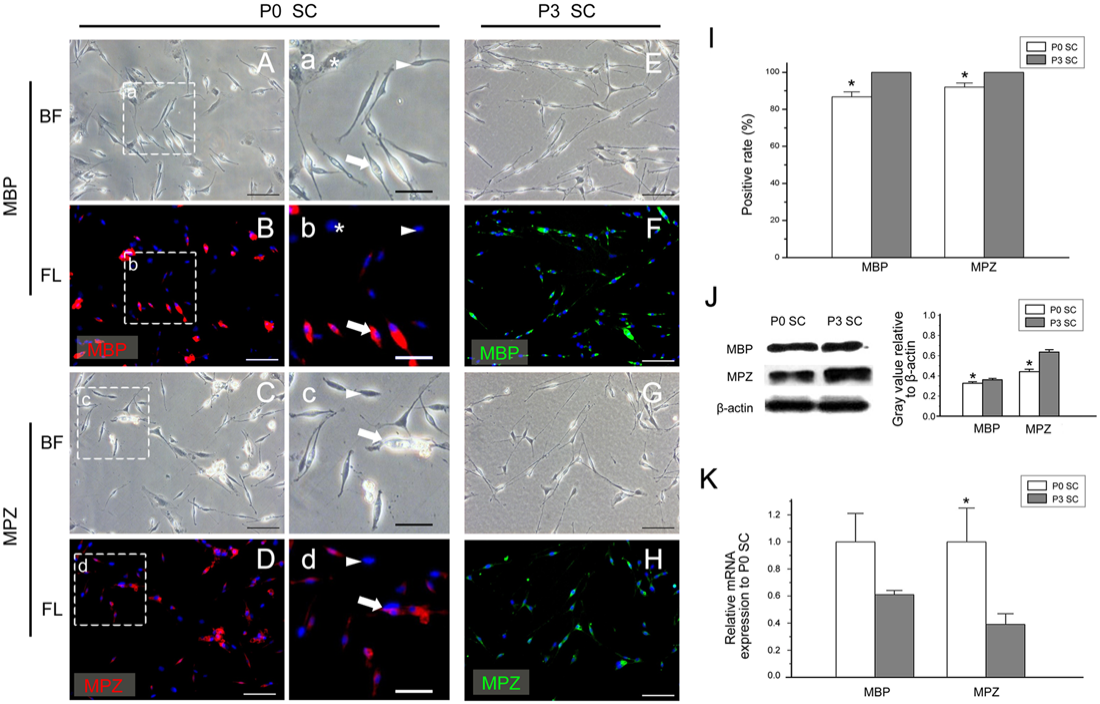
The expression of MBP and MPZ in cultured SCs. (A-H, a-d) The expression of MBP and MPZ in *in vitro* cultured SCs detected by immunofluorescence. In primary cultures (P0), most SCs were MBP or MPZ positive (a-d arrow) with strong refraction rates and translucent cell bodies; a small part of the SCs (a-d arrowhead) and all of the fibroblasts (a, b asterisk) were MBP- or MPZ-negative with poor refraction rates and halos around the cells. In the third passage (P3),all SCs expressed MBP and MPZ (E-H). The bright-field (A, C, E, G, a, c) and the immunofluorescence channels (B, D, F, H, b, d) are displayed. (I) The positive expression rates of MBP and MPZ in P0 SCs and P3 SCs. (J) The protein levels of MBP and MPZ in P0 SCs and P3 SCs were detected by Western blotting. The protein levels of both markers were higher in P3 SCs than those in P0 SCs. (K) The mRNA levels of MBP and MPZ were determined by real-time quantitative RT-PCR analysis. The mRNA level of MPZ was higher in P0 SCs than that in P3 SCs, while no significant difference of MBP’s mRNA level was detected. (A-H) bar = 100μm, (a-d) bar = 50μm. * *P*<0.05.

Transcription factor Oct6 interacts with transcription factor Krox20 to regulate myelination and affects the expression of myelin-specific proteins MBP and MPZ in mSC [5,38–41]. Therefore, we further examined the expression of MBP and MPZ in *in vitro* cultured SCs. At the early stage of cultured SCs, the positive expression rates of MBP and MPZ were 86.74% ± 2.66% and 91.95% ± 2.25%, respectively, and both of them reached 100% in P3 SCs (Fig 5I). In addition, we observed that MBP-positive SCs or MPZ-positive SCs were generally highly translucent, and those cells are transparent when observed under the light microscope (Fig 5a–5d arrow). However, SCs with negative or low expression of MBP or MPZ had poor transparency, dark cell bodies and visible nuclei. Halos appear at the outer edge of the membranes of these cells (Fig 5a–5d arrowhead), which differs from the typical morphology of fibroblasts (Fig 5a and 5b asterisk).

### The expression of NCAM and GAP43

**Fig 6:**
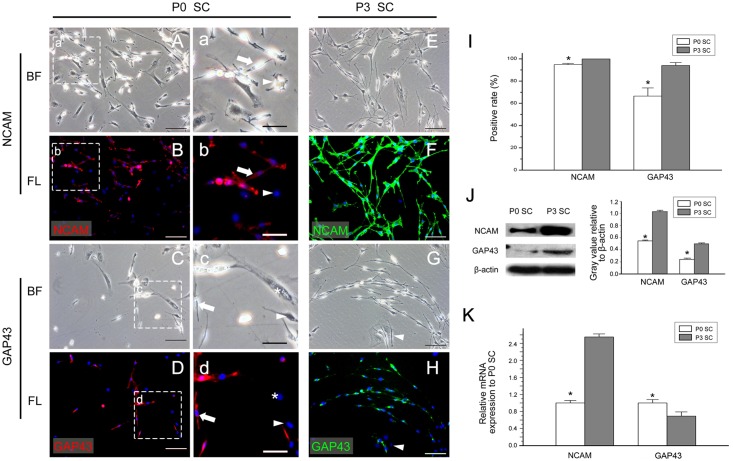
The expression of NCAM and GAP43 in cultured SCs. (A-H, a-d) The expression of NCAM and GAP43 in *in vitro* cultured SCs detected by immunofluorescence. Most SCs in P0 SCs were NCAM and GAP43 positive (a-d arrow). A small portion of SCs (a-d arrowhead) and all fibroblasts (a-d asterisk) were NCAM and GAP43 negative. All SCs in P3 expressed NCAM (E, F), but a small portion of SCs were GAP43 negative (G, H arrowhead). The bright-field (A, C, E, G, a, c) and the immunofluorescence channels (B, D, F, H, b, d) are displayed. (I) The positive expression rates of NCAM and GAP43 in P0 SCs and P3 SCs. (J) The protein levels of NCAM and GAP43 in P0 SCs and P3 SCs were detected by Western blotting. (K) The mRNA levels of NCAM and GAP43 in P0 SCs and P3 SCs were determined by real-time quantitative RT-PCR analysis. (A-H) Scale bar = 100μm, (a-d) Scale bar = 50μm. * *P* <0.05.

Besides mSC, iSC can differentiate into nmSC as well. The *in vivo* markers of iSC and nmSC are NCAM and GAP43, which are very similar [6,19,42]. Through our immunofluorescence experiment, we found that most of the early culture SCs expressed NCAM at the positive rate of 94.75% ± 1.19% (Fig 6I). Interestingly, although GAP43 is expressed in iSC and nmSC *in vivo*, its *in vitro* expression pattern was similar to Oct6’s expression, which was not fully expressed in all *in vitro* SCs. GAP43 showed the positive rate of 66.60% ± 7.30% and 94.06% ± 2.62% at early and later culture time points, respectively (Fig 6I).

### Potential multiple SC stages in newborn mice

By immunofluorescence, we detected nine SC markers, except for Sox2, expressed in SN in newborn mice. For technical reasons, we could not examine the expression rate of these SC markers *in vivo* in the present study. However, previous studies have demonstrated that transcription factor Sox2 is only transiently expressed in SCPs and iSCs of mouse embryos before myelination, and its expression level rapidly decreases after birth [5,28,29]. That may be the reason that Sox2 was not detected in SN harvested from either newborn mice or adult mice. Another report showed that the expression of transcription factor Oct6 peaks on day 0 after birth, followed by gradual decreasing, and no expression in mature mSCs [37,43]. Consistent with these previous reports, we detected expression of Oct6 in the newborn mice, but not in the adult mice. Based on our finding and previous reports, we speculated that in newborn mice SC might exist as iSC, pro-mSC, mSC and nmSC; while in adult mice SCs exist as the two major mature stages, which are nmSC and mSC.

### S100 is not a reliable pan-SC marker

S100 is the most commonly used SC marker, and prior studies suggest that S100 is expressed in iSC, pro-mSC, mSC and nmSC *in vivo*, but not in SCP [37,44–46]. Therefore, we first examined the expression of S100 in *in vitro* cultured SC. We found that, in early culture, only about 85% of the SCs expressed S100, while all SCs cultured for eight days expressed S100. Some studies show that S100 is closely related to the maturation of SC [45,47], and therefore we interpreted our data as reflecting that in SN of newborn mice, a considerable portion of SCs are in SCP stage, which do not express S100. Then, after a period of time *in vitro*, the environment and various cytokines secreted by SCs, such as BDNF and GDNF, may promote SCP to differentiate into iSC [48,49].

Real-time quantitative RT-PCR results showed that, in the late stage of *in vitro* cultured SCs, the mRNA level of S100 was unexpectedly lower than that in the earlier cultures with unknown reason. We hypothesized that these SCs may be in an immature stage, and S100 expression in these cells was negatively regulated, which led to a low mRNA level.

Because S100 is not fully expressed in the early cultured SCs, and because a variety of other cells, such as chondrocytes, adipocytes, cardiomyocytes, skeletal muscle cells, and melanoma cells, also express S100 *in vivo* [45,50,51], we believe that S100 alone cannot be used as a marker to identify cultured SCs. Moreover, S100 expression in induced SCP is negative, but S100 expression can be positive if the induced cells contain other S100-positive cells. Thus, S100 cannot be used singly to identify SC-like cells derived from various types of stem cells either.

### Sox10 expression is necessary for SC identification

Transcription factor Sox10 is a specific marker for neural crest-derived cells, and it is expressed in neural crest cells and various types of differentiated cells, including all types of SCs. Sox10, a key transcription factor, is essential for the development of SCs [52], and is involved in the regulation of myelination [19]. In this study, *in vitro* cultured SCs always express Sox10, which is similar to the *in vivo* expression pattern. Possibly the expression of Sox10 is not easily affected by environmental changes. These results suggest that Sox10 may be used to identify *in vitro* cultured SCs. However, Sox10 alone cannot distinguish SCs in different types or different developmental stages, and it cannot distinguish between other cell types derived from neural crest cells, which suggests that it cannot be used singly to identify SC-like cells after stem-cell induction. Since Sox10 is stably expressed *in vitro* and *in vivo*, it could serve as a necessary, but not sufficient, marker for identifying SC and SC-like cells.

### The phenotype of *in vitro* cultured SC is similar to that of iSC

Transcription factor Sox2, which is in the same family as Sox10, is expressed in undifferentiated multipotent cells in various tissues, including neural stem cells [53]. After these progenitor cells differentiate, the Sox2 level decreases, indicating that Sox2 plays a very important role in maintaining cells in an undifferentiated state [54,55]. In addition, Sox2 is a marker of stem-cell pluripotency in a variety of tissues [28,56,57]. A previous study has found that Sox2 is first expressed in undifferentiated SC in uninjured SN, and that its level significantly increases in dedifferentiated SCs during the process of Wallerian degeneration after nerve crush [5].

In this study, Sox2 is not significantly expressed in SCs in neonatal mouse SN, but it is expressed in most cultured SCs. This suggests that *in vivo* SCs are mainly in the mature state (mSC or nmSC) in newborn mice, while the majority of *in vitro* SCsmay be in iSC state. A possible explanation might be that *in vitro* SCs lose contact with axons, leading to rapid dedifferentiation from mature state into iSC, a process similar to the *in vivo* Wallerian degeneration process.

Our data also showed that the majority of *in vitro* cultured SCs express GAP43, P75^NTR^ and NCAM. Since these markers are usually expressed in nmSC in normal nerves or in iSC in injured nerves [58], it is hard to tell whether *in vitro* cultured SCs were derived from nmSCs or iSCs. However, previous studies have found a clear distinction between nmSCs and iSCs: MPZ mRNA expression is not found in mature nmSCs [59]. Thus, the mRNA level of MPZ can be used to distinguish between these two states of SCs. Using real-time quantitative RT-PCR assay, we found that the mRNA level of MPZ in the early cultured SCs was much higher than that in the late-culture ones, suggesting that *in vitro* cultured SCs, especially after a long culture, were much closer to iSCs than to nmSCs.

Of note, previous studies show that *in vivo* iSCs do not express Krox20 and MBP. However, our data show that with increased incubation time the ratio of Krox2-positive and MBP-positive SCs gradually reached 100%, suggesting that *in vitro* cultured SCs are similar, but not identical, to *in vivo* iSC.

The difference of SC marker expression between *in vivo* and *in vitro* was summarized in Table 2.

**Table 2:** Comparison of markers expression between *in vivo* and *in vitro* Schwann cells.

Markers	S100	Sox10	Oct6	Krox20	Sox2	P75^NTR^	NCAM	GAP43	MBP	MPZ
Adult SN	(+)	(+)	(-)	(+)	(+)	(+)	(+)	(+)	(+)	(+)
Newborn SN	(+)	(+)	(+)	(+)	(+)	(+)	(+)	(+)	(+)	(+)
P0 SCs	85.84% ± 1.86%	100%	82.60% ± 4.13%	91.67% ± 4.13%	100%	93.57% ± 3.40%	94.75% ± 1.19%	66.60% ± 7.30%	86.74% ± 2.66%	91.95% ± 2.25%
P3 SCs	100%	100%	92.64% ± 3.49%	100%	100%	100%	100%	94.06% ± 2.62%	100%	100%

## Conclusions

In conclusion, our data drew an elementary marker map of SCs cultured *in vitro* and showed that multiple SC stages may exist *in vivo* in neonatal mice. For SC identification, S100 is not a very reliable marker, while Sox10 is a necessary but not sufficient marker. *In vitro* cultured SCs express Sox2, P75^NTR^, NCAM, GAP43, Oct6 and MPZ, and their state is similar to *in vivo* undifferentiated iSC and dedifferentiated iSC in damaged nerves.
